# Demographic and need factors of early, delayed and no mental health care use in major depression: a prospective study

**DOI:** 10.1186/s12888-017-1531-8

**Published:** 2017-11-16

**Authors:** A. M. Boerema, M. ten Have, A. Kleiboer, R. de Graaf, J. Nuyen, P. Cuijpers, A. T. F. Beekman

**Affiliations:** 1 Department of Clinical Neuro and Developmental Psychology, Section clinical psychology, Faculty of Behavioural and Movement Sciences, Vrije Universiteit Amsterdam, Amsterdam, the Netherlands; 2 EMGO+ institute for Health Care and Research, VU Medical Centre, van der Boechorststraat 7, 1081 BT Amsterdam, The Netherlands; 3 Netherlands Institute of Mental Health and Addiction, Da Costakade 45, 3521 VS Utrecht, The Netherlands; 4 Department of Psychiatry, VU Medical Centre, van der Boechorststraat 7, 1081 BT Amsterdam, The Netherlands

**Keywords:** Depression, Mental health care use, Delayed treatment, No treatment, Early treatment, Symptom severity

## Abstract

**Background:**

Despite the availability of evidence based treatments, many people with major depression receive no or delayed professional treatment, which may put them at risk for adverse outcomes. The aim of this study was to examine which demographic and need factors distinguish early, delayed and no treatment use.

**Methods:**

Data were obtained from the Netherlands Mental Health Survey and Incidence Study-2 (NEMESIS-2). People with a diagnosis of major depression in the past 12 months were included (*N =* 434). Mental health care use was assessed during this same period and at follow up (three years later). Multinomial regression analysis was used to distinguish early, delayed and no mental health care users with respect to demographic and need factors.

**Results:**

The majority of participants accessed treatment early (62%). Early treatment users were characterized by more severe and persistent symptoms and were more likely not to have a partner compared to no treatment users. The majority of those without treatment reached remission in three years (85%). Delayed treatment users were, compared to early users, characterized by relatively mild symptoms and a persistent or new major depressive disorder at follow up.

**Conclusions:**

Early access to treatment and the finding that need factors determine mental health care use among people with depression show that the filters along the pathway to treatment are not influenced by unfavorable determinants like education or age.

## Background

Major depression is a highly prevalent mental disorder [1] and is associated with substantial personal and economic burden [2–5]. Despite the availability of evidence-based psychological and pharmacological therapies, a substantial number of people with depression do not seek or receive mental health care [6–9]. In addition, a delay in mental health care use for depression is reported in several studies [10–13]. Attitudinal barriers, such as the desire to handle problems by themselves [14] or the belief that the depressive symptoms will abate spontaneously [15] have shown to be related with delayed or no treatment use. In some cases this might be justified, as findings show that about 50% of the people with first episodes of major depression in the general population recover within 3 months [16]. However, recovery rates decline rapidly after this period [16], implying an increased risk of adverse outcomes when people postpone or put off seeking help [17]. For this reason, it is important to examine which factors are associated with delayed or no mental health care use.

Cross-sectional epidemiologic studies have shown that increased mental health care use is associated with having more severe symptoms and a comorbid mood or anxiety disorder [9, 18–21]. In addition, findings showed that people with a mood and/or anxiety disorder in the past 12 months who reported more functional impairments on several areas (e.g. work, household, social contacts), were more likely to use mental health care [20]. Several attitudinal and demographic characteristics are associated with mental health care use as well [22–24]. People with low perceived social support, who recognize themselves as having mental problems and people who perceive a need for care are more likely to use mental health care [24–27]. Demographic variables associated with increased mental health care use are being female, age and not having a partner [6, 28, 29]. Population-based research on treatment delay in people with common mental disorders has shown that men, older age groups and people who report a younger age at onset of their disorder report longer treatment delays [10, 11]. A longitudinal population study in the Dutch general population examined mental health care use in people who were diagnosed with a mood and/or anxiety disorder in the preceding 12 months at baseline [26]. People were followed for three years and factors related to mental health care use were examined. The results showed that people who reported having a persistent or new episode between baseline and follow up (after three years), people with suicidal thoughts and people who feel comfortable with professional help, were more inclined to use professional health care after three years [26].

In summary, based on earlier research findings from cross sectional studies, delayed treatment users may be distinguished from early treatment users by several demographic (gender or age) and need factors (severity of symptoms). However, most knowledge is derived from cross-sectional designs which preclude the investigation of long term associations. Large representative longitudinal population studies regarding mental health care use are rare [26, 30–32]. Consequently, knowledge about determinants that influence mental health care use in delayed treatment users is limited. Furthermore, a longitudinal design provides the opportunity to examine mental health care use over time, especially in relation to need factors (e.g. persistence of symptoms and/or new major depressive disorder). For example, results from two Dutch longitudinal population studies indicate that the majority of people (50%–80%) who do not receive mental health care reached remission after a certain period [26, 30, 32, 33], suggesting that for some people the problems were probably self-limiting. Moreover, two cross-sectional analyses based on population studies showed that the majority of people with a mood disorder (about 80%) in the general population will eventually make contact with mental health care at some point during their life [10, 11] indicating that a distinction between early, delayed and no treatment users is important. However, to our knowledge, only one study made such a distinction among people with common mental disorders [26] and one study among people with alcohol use disorder [34]. For these reasons, more research regarding mental health care use in a large representative sample of the general population is necessary.

The aim of the present study is to examine whether early, delayed and no treatment users differ regarding demographic (gender, age, education, partner and work status) and need factors (comorbid anxiety disorder, duration of the depressive episode, disability days at work or normal activities, new or persistent major depressive disorder and severity of symptoms). For the present paper we used data from the Netherlands Mental Health Survey and incidence Study-2 (NEMESIS-2). This general population study has a longitudinal design which improves understanding of mental health care use over time.

## Methods

### Sample

The primary aim of NEMESIS-2 was to provide up- to- date information on the prevalence, incidence and course of DSM-IV mental disorders in the general population and its consequences in terms of service use and functioning. NEMESIS-2 is a longitudinal study which incorporated 3 waves (baseline 2007–2009; follow up T_1_ 2010–2012; follow up T_2_ 2013–2015). The methods employed have been described in greater detail elsewhere [35]. In a multistage, stratified random sampling procedure, 184 of the 443 existing municipalities were drawn. In these municipalities, a random sample of addresses of private households from postal registers was taken. Based on the most recent birthday at first contact within the household, an individual aged 18–64 years with sufficient fluency in the Dutch language was randomly selected for a face-to-face interview [36]. The study was approved by a medical ethics committee (the Medical Ethics Review Committee for Institutions on Mental Health Care, METIGG). After having been informed about the study aims, respondents provided written informed consent. The response rate of the baseline measurement was 65.1% and consisted of 6646 respondents. The sample was nationally representative, although younger subjects were somewhat underrepresented [35]. All 6646 baseline participants were approached for first follow-up (T_1_) three years after baseline (2010–2012), of which 5303 could be reinterviewed (80.4% response). All these 5303 participants were approached for second follow (T_2_) up three years after T_1_ (2013–2015), of which 4618 could be reinterviewed (87.8% response) [37]. Attrition at T_1_ and T_2_ was not significantly associated with all individual 12-month mental disorders at baseline (controlled for sociodemographic factors) [36, 37].

### Subjects of the present study

From the 6646 subjects that were included during the baseline measurement, people with a diagnosis of major depression in the preceding 12 months at the baseline measurement (T_0_) were selected, and we assessed whether they did or did not use primary care or mental health care for their psychological problems in this same period and at follow up (three years) (T_1_). Because of this relatively small subsample (*n* = 287) we also made the same selection of respondents in the second wave (T_1_ and T_2_): we selected people (who were not selected at baseline as described above) with a diagnosis of major depression 12 months prior to T_1_ and assessed whether they did or did not use primary care or mental health care for their psychological problems at T_1_ and follow up (T_2_)_._ Since we used the same selection criteria, the data could be pooled and the analysis could be performed on the whole group. This resulted in 434 respondents (N_T0-T1_ = 287, N_T1-T2_ = 147).

### Composite international diagnostic interview (CIDI)

DSM-IV disorders were assessed using the Composite International Diagnostic Interview (CIDI) 3.0, which was developed in the World Mental Health Survey (WMHS) Initiative [35, 38–40]. The CIDI 3.0 version used in NEMESIS-2 was an improvement on the Dutch one used in this initiative. For the present study the depression section of the CIDI 3.0 was used to determine the presence of a major depression. Comorbidity with any anxiety disorder (panic disorder, agoraphobia (without panic disorder), social phobia, specific phobia, generalized anxiety disorder (GAD)) was determined with the CIDI 3.0 interview as well. The CIDI 3.0 interview was conducted at each wave and for this paper we used the 12 months prevalence of above mentioned mental disorders at every wave (baseline, follow-up). Clinical calibration studies conducted in various countries have found that CIDI 3.0 assesses anxiety and mood disorders with generally good validity compared to blinded clinical reappraisal interviews [41].

### Mental health care use

At baseline, mental health care use was measured with the following question: ‘In the previous 12 months, have you visited any of the following professionals or institutions for emotional problems or alcohol or drugs problems of your own?’ and at follow up: ‘Since the last interview, did you visit any of the following professionals or institutions because of emotional or alcohol or drugs problems of your own?’ Primary care included general medical professionals (general practitioners, company doctors, social work, home care or district nurses, physiotherapists or haptonomists, medical specialists or other professionals working within the general medical sector). Specialized mental health care included psychiatrists, psychologists, psychotherapists, mental health care or addiction institutes, part-time or full-time psychiatric treatment [20, 35].

Mental health care use refers to at least one contact made in primary care and or specialized mental health care for emotional or addiction problems in the past 12 months prior to baseline measurement (T_0_). For the people that were selected at T_1_, mental health care use was assessed 12 months prior to T_1._ At follow up, we assessed if people report any mental health contact between the two successive measurements (T_0_-T_1_ or T_1_-T_2_).

Three groups of mental health care users were distinguished:

1. *No* treatment users (people who did not report any contact with primary or specialized mental health care in the past 12 months prior to the first measurement and at follow up (T_0_-T_1_ or T_1_-T_2_, in total 4 years);
2. *Delayed* treatment users (people who did not report any contact with primary care or specialized mental health care contact in the past 12 months before the first measurement (T_0_ or T_1_) but who reported contact at follow up (T_1_ or T_2_, in the following 3 years);
3. *Early* treatment users (people who reported primary or specialized mental health care contact in the past 12 months before the first measurement (T_0_ or T_1_)).

### Predictors of service use

Potential predictors of mental health care use were recorded at baseline for the T_0_-T_1_ cohort and at T_1_ for the T_1_-T_2_ cohort. The predictor variables included the following:

Demographic factors.

*Gender* categorized into male and female;

*Age* categorized into 18–44 years old or 45–66 years old (categorization based on frequency pattern, 0 = 49%, 1 = 51%);

*Education* defined as primary education/lower secondary education; higher secondary education; higher professional education, university.

*Partner status* defined as partner or no partner;

*Job status* defined as having a job or no job;

Need factors.

*Comorbid anxiety disorder:* This variable relates to comorbidity with any anxiety disorder in the past 12 months before the T_o_ or T_1_ measurement with the CIDI 3.0.

*Disability days on work or normal daily activities* based on the CIDI 3.0 question ‘About how many days out of 365 in the past 12 months were you totally unable to work or carry out your normal activities because of your (sadness/or/discouragement/or/lack of interest)’.

We categorized this variable into 0 = ≤ 2 weeks and 1 = ≥ 2 weeks (categorization based on frequency pattern, 0 = 55%, 1 = 45%).

*Duration depressive episode in past 12 months* based on the CIDI 3.0 question ‘About how many days out of the last 365 were you in an episode?’ We categorized this question in: 0 = ≤ 3 months and 1 = ≥ 3 months (categorization based on frequency pattern, 0 = 49%, 1 = 51%).

*Severity of the depressive symptoms* was measured with the Inventory of Depressive Symptomatology, shortened version (QIDS-sr_16_) in the CIDI 3.0 [42]. The aim of this questionnaire is to assess the severity of depressive symptoms. The total scores range from 0 (none) until 27 (very severe). For the present study we categorized this variable into 3 categories: none/mild (0–10); moderate (11–15); severe/very severe (21–27) (categorization based on the QIDS-sr_16_ manual).

*Major depression at follow-up measurement* this variable related to a persistent or new 12-month major depression at follow-up measurement (T_1_ or T_2_), measured with the CIDI 3.0.

### Statistical analyses

The data from the two waves (T_0−_T_1_ and T_1−_T_2_) were merged into one dataset. In the final dataset (e.g. the dataset we used to perform the analyses), there were no duplicates in the selected respondent of T_0_ and T_1_. If respondents were both in the group at T_0_-T_1_ and T_1_-T_2,_ they were excluded from the T_1_-T_2_ cohort.

A multinomial logistic regression analysis was performed to determine the effects of demographic and need factors on mental health care use among those with a 12-month major depression at first measurement (T0 or T1). The dependent variable, primary care or mental health care use, consisted of three categories: early treatment use, delayed treatment use, no treatment use. Early treatment use was used as the reference category, because delayed and no treatment users were of specific interest.

We conducted a series of univariate analyses for each predictor separately (unadjusted odds ratios (OR)). Then, a multivariate analysis was performed to determine the effect of individual predictors in which all predictors were entered simultaneously (adjusted OR). The regression analyses were performed using SPSS 21 and a two sided significance level of *p* < .05 was used in all analyses.

In order to adjust for possible differences between the two waves (T_0_ -T_1_ or T_1_-T_2_), we added an identification variable for the individual cohorts in all regression models (dummy variable study ‘cohort’). The identification variable was significant in the comparison between the no treatment users and early treatment users (*p* = .03), but not in the comparison between delayed and early treatment users (*p* = .11). Therefore, a sensitivity analysis was conducted were the analyses were performed separately on the T_0_ -T_1_ (*N* = 287) or T_1_-T_2_ (*N* = 147) study cohorts. These data were compared with the results of the total group (*N* = 434). This sensitivity analysis revealed no substantial differences between the two cohorts (results available on request). Furthermore, we performed the multinomial regression analyses with or without the identification variable. The results from these analyses revealed no substantial differences between the analyses as well (results available on request). Since the sensitivity analyses did not reveal substantial differences, but the variable was significant in the no treatment users versus early treatments users we performed the analyses on the dataset including the identification variable.

Missing values were present for three variables; disability days on work and/or normal daily activities (10,8%, *n* = 47), duration depressive episode in past 12 months (10,6%, *n* = 45) and severity of depressive symptoms (6,9%, *n* = 30). In order to adjust for the missing data we imputed data for these variables in the dataset using a Fully Conditional Specification (MCMC) model with a Predictive Mean Matching (PMM) model type and 1E-012 as singularity tolerance and *N* = 10 imputations. Sensitivity analyses on the original dataset and imputed dataset yielded no significant differences (results available on request). Therefore, the analyses were performed on the imputed dataset.

## Results

### Characteristics of the study sample

The majority of the 434 respondents with a 12-month major depression at ‘first’ measurement, were female (67%). Respondent were, on average, 44 years old (range 18–67 year) at baseline. Most respondents (39%) completed higher secondary education or primary education/ lower secondary education (33%). About half (48%) reported living with a partner (Table 1).

**Table 1:** Baseline characteristics associated with early, delayed and no treatment users among people with 12-months MDD

	Overall population (*N =* 434)	Early treatment users (*N =* 271, 62%)	Delayed treatment users (*N =* 58, 14%)	No treatment users (*N =* 105, 24%)
	*N* (%)	*n* (%)	*n* (%)	*n* (%)
*Socio-demographic factors* ^*a*^				
Female	291 (67)	182 (67)	37 (64)	72 (69)
Older age (45–66 years old)	220 (51)	139 (51)	25 (43)	56 (53)
Education				
primary/lower secondary education	145 (33)	84 (30)	18 (31)	43 (41)
higher secondary education	168 (39)	107 (40)	23 (40)	38 (36)
higher professional education, university	121 (28)	80 (30)	17 (29)	24 (23)
No partner	207 (48)	137 (51)	33 (57)	37 (35)
No job	151 (35)	101 (37)	15 (26)	35 (33)
*Need factors* ^*a*^				
Comorbid anxiety disorder	151 (35)	113 (42)	21 (36)	17 (16)
Longer duration episode in past 12 months (>3 months) ^c^	220 (51)	149 (55)	29 (50)	42 (40)
Disability days on work and/or normal daily activities (>2 weeks) ^c^	190 (44)	140 (52)	18 (31)	32 (30)
Major depressive disorder between baseline and follow-up ^b c^	123 (28)	84 (31)	25 (43)	14 (13)
Severity depressive symptoms (IDS)				
mild	78 (18)	32 (11)	17 (29)	30 (28)
moderate	135 (31)	75 (28)	16 (28)	43 (42)
severe/very severe	221 (51)	164 (61)	25 (43)	32 (30)
*Other variables*				
Mental health care use at follow-up ^*b*^				
none	212 (49)	107 (40)	0 (0)	105 (100)
primary care only	61 (14)	31 (11)	30 (52)	0 (0)
specialized mental health care only	23 (5)	19 (7)	4 (7)	0 (0)
primary + specialized mental health care	138 (32)	114 (42)	24 (41)	0 (0)
Major depressive disorder at follow up (past 12 months) ^*b*^	86 (20)	61 (23)	14 (24)	11 (11)

^a^Determinants recorded at baseline

^b^Determinant recorded between baseline and first measurement for the first cohort (T0-T1) and between T1-T2 for the second cohort

### Mental health care use

The majority of the respondents with major depression in the 12 months prior to the first measurement (62%, *n =* 271) reported mental health care use in the same period (T_0_ or T_1_) (early treatment users). About a quarter (24%, *n =* 105) of the respondents reported no mental health care use at follow up (T_0_ -T_1_ or T_1_-T_2_) (no treatment users). Finally, 14% (*n =* 58) of the respondents did not report mental health care use at first measurement (T_0_ or T_1_), but did three years later (delayed treatment users) (Table 1).

### Major depressive disorder at follow up (T_0_-T_1 or_ T_1−_T_2_)

About a quarter of the early and delayed treatment users reported a major depressive disorder at follow up, respectively 23% (*n* = 61) and 24% (*n* = 14). Among the no treatment users 11% (*n* = 11) reported a depressive disorder at follow up.

### No treatment users versus early treatment users

Univariate analyses showed that no treatment users could be distinguished from early treatment users by several factors related to need for care (Table 2). People who reported mild to moderate symptoms, no comorbid anxiety disorder, a shorter depressive episode (< 3 months), no persistent or new major depressive disorder between T_0_-T_1_ or T_1_-T_2_ and less disability days (< 2 weeks), were more likely to be in the no treatment group. People without a partner were more likely to receive mental health care in the 12 months prior to the first measurement.

**Table 2:** Determinants of mental health care service utilization (reference category dependent variable: early treatment users)

	No treatment users ^*a*^			
	Univariate analyses		Multivariate analyses	
	Unadjusted OR ^d^ (95% CI ^e^)	*p*	Adjusted OR ^d^ (95% CI ^e^)	*p*
*Socio-demographic factors* ^*b*^				
Male	0.95 (0.58–1.54)	0.83	0.92 (0.54–1.59)	0.78
Younger age (18–44 years old)	0.90 (0.57–1.42)	0.66	0.95 (0.56–1.59)	0.84
Education (ref. higher professional education, university)				
primary/lower secondary education)	1.63 (0.90–2.94)	0.11	1.68 (0.87–3.26)	0.12
higher secondary education	1.21 (0.67–2.19)	0.53	1.29 (0.68–2.45)	0.44
Partner	2.11 (1.31–3.40)	0.002	1.85 (1.60–3.13)	0.02
Job	1.21 (0.75–1.96)	0.43	0.86 (0.49–1.50)	0.59
*Need factors* ^*b*^				
No comorbid anxiety disorder	4.23 (2.36–7.59)	0.000	3.18 (1.70–5.95)	0.000
Shorter duration episode past 12 months (<3 months)	1.75 (1.08–2.48)	0.02	1.32 (0.78–2.22)	0.30
Disability days on work and/or normal daily activities (<2 weeks)	2.29 (1.36–3.86)	0.002	1.57 (0.88–2.80)	0.13
Major depressive disorder between baseline and follow-up ^c^	0.36 (0.19–0.66)	0.001	0.43 (0.22–0.84)	0.01
Severity depressive symptoms (ref. (very) severe)				
none/mild	4.67 (2.45–8.97)	0.000	2.45 (1.19–5.06)	0.02
Moderate	2.89 (1.67–4.99)	0.000	2.37 (1.30–4.31)	0.005
*P* for trend		<0.000		0.03

In order to adjust for possible differences between the different cohorts, we added an identification variable for these cohorts in the regression models

^a^Reference category dependent variable: early treatment users

^b^Determinants recorded at baseline

^c^Determinant recorded between baseline and first measurement for the first cohort (T0-T1) and between T1-T2 for the second cohort

^d^ *OR* Odds ratio

^e^ *CI* Confidence Interval

In the multivariate analyses the effect for having a partner, no comorbid anxiety disorder mild to moderate symptoms (as compared to early treatment users) and no persistent or new major depressive disorder between T_0_-T_1_ or T_1_-T_2_ remained significant (Table 2). The univariate effect of disability days and shorter episode duration lost significance in the multivariate analyses. A possible explanation may be that these factors correlate with severity of symptoms, which is a stronger predictor of treatment use.

### Delayed treatment users versus early treatment users

Univariate analyses showed that delayed treatment users could be distinguished from early treatment users by two need for care factors. People who reported <2 weeks of disability days and people who report none to mild symptoms (as compared to early treatment users) were more likely to be in the delayed treatment group compared to early treatment users (Table 3).

**Table 3:** Determinants of mental health care service utilization (reference category dependent variable: early treatment users)

	Delayed treatment users ^*a*^			
	Univariate analyses		Multivariate analyses	
	Unadjusted OR ^d^ (95% CI ^e^)	*p*	Adjusted OR ^d^ (95% CI ^e^)	*p*
*Socio-demographic factors* ^*b*^				
Male	1.17 (0.65–2.12)	0.60	1.09 (0.58–2.04)	0.80
Younger age (18–44 years old)	1.37 (0.77–2.43)	0.28	1.26 (0.69–2.32)	0.45
Education (ref. higher professional education, university)				
primary/lower secondary education	0.97 (0.47–2.02)	0.94	1.11 (0.50–2.46)	0.80
higher secondary education	1.03 (0.52–2.06)	0.93	1.13 (0.54–2.34)	0.75
Partner	0.83 (0.47–1.49)	0.54	0.78 (0.42–1.46)	0.43
Job	1.73 (0.91–3.28)	0.09	1.61 (0.80–3.25)	0.18
*Need factors* ^*b*^				
No comorbid anxiety disorder	1.38 (0.76–2.52)	0.29	1.13 (0.58–2.20)	0.71
Shorter duration episode past 12 months (<3 months)	1.16 (0.63–2.13)	0.64	0.92 (0.48–1.77)	0.82
Disability days on work and/or normal daily activities (<2 weeks)	2.37 (1.22–4.60)	0.01	1.85 (0.91–3.75)	0.09
Major depressive disorder between baseline and follow-up ^c^	1.69 (0.97–3.21)	0.06	2.19 (1.16–4.13)	0.02
Severity depressive symptoms (ref. (very) severe)				
none/mild	3.33 (1.57–7.10)	0.002	3.23 (1.41–7.38)	0.005
moderate	1.35 (0.65–2.77)	0.41	1.21 (0.57–2.59)	0.62
*P* for trend		0.003		0.02

In order to adjust for possible differences between the different cohorts, we added an identification variable for these cohorts in the regression models

^a^Reference category dependent variable: early treatment users

^b^Determinants recorded at baseline

^c^Determinant recorded between baseline and first measurement for the first cohort (T0-T1) and between T1-T2 for the second cohort

^d^ *OR* Odds ratio

^e^ *CI* Confidence Interval

In the multivariate analyses the effect for none to mild symptoms remained, while the univariate effect of disability days disappeared in the multivariate analyses (Table 3). Furthermore, the effect of a persistent or new major depressive disorder was only significant in the multivariate analyses, possibly due to the relative small sample size (e.g. the results are in the same direction, *p =* 0.06 for the univariate analyses *p =* 0.02 for the multivariate analyses) (Table 3).

### Delayed treatment users versus no treatment users

By changing the reference category in the multinomial regression analyses, we examined demographic and need factors between delayed and no treatment users as well. Multivariate analyses showed that delayed treatment users, compared to no treatment users, were more likely to have a major depressive disorder between baseline and follow-up (OR = 5.08, CI==2.23–11.57, *p* = <.001) and a comorbid anxiety disorder (OR = 2.88, CI = 1.26–6.66, *p* = .02). Furthermore, delayed treatment users were less likely to have a partner (OR = 0.42, CI = 0.21–0.86, *p* = .02).

## Discussion

Although there are several evidence based effective treatments for depression and although in countries like the Netherlands access to treatment is relatively good [43, 44], research showed that from the people with a 6-month major depressive disorder at baseline more than half (54%) did not use mental health care one year later [33]. However, research findings revealed as well that the majority of people (about 80%) in the general population with a mood disorder will eventually make contact with mental health care at some point *during their life* [10, 11]. For this reason, we aimed to examine which demographic (gender, age, education, partner and job status) and need factors (comorbid anxiety disorder, duration depressive episode, new or persistent major depressive disorder and severity of symptoms) distinguish early, delayed and no treatment users among those with a 12-month DSM-IV major depression in the general population.

The results showed that the majority of people with a major depressive disorder in the past 12 months received mental health care in the same period (62%). Early treatment users were categorized by more severe and persistent depressive symptoms. The majority of the no treatment users reached remission after three years (85%), which suggests that they were able to solve their problems without professional help or that the depressive symptoms were self-limiting. This high remission rate among no treatment users is in line with earlier research findings in the Netherlands [26, 32, 33]. From the demographic factors, having a partner was associated with no treatment use. Also other studies showed that having a partner is associated with decreased mental health care use [6, 28, 29]. In addition, there are some indications that access to social support is associated with less treatment use [27, 45] and being able to reach remission without professional help [26, 46, 47].

In the present study the group of delayed treatment users was relative small (14%) and they were, compared to early treatment users, more likely to have mild depressive symptoms and to report a persistent or 12-month depression after three years [15, 20, 26]. High illness severity has shown to be a prompt reason for treatment use in people with an initial treatment delay [15, 19]. Possibly, delayed treatment users will use mental health care when depressive symptoms do not abate or when a depressive disorder becomes apparent.

Except for having a partner in the group of no treatment users, no other demographic factors were associated with early, delayed or no treatment use. A possible explanation is that we focused our analyses on recent treatment use. Other studies examined life-time treatment contact or examined the time between the onset of symptoms and first treatment contact, which could explain possible differences [10, 11].

The study results suggest that the accessibility of the mental health care system for major depression in the Netherlands is relatively good (>60% early users) and largely determined by clinical need for care related factors, such as severity of symptoms. Delayed or no treatment users are characterized by less severe and less complex symptoms compared to early treatment users. The fact that demographic factors (except for partner status in the no treatment users) were not associated with treatment use may be a positive result, since people should not receive help based on their level of education, gender or age. Not everyone from the no treatment users group reached remission after three years (11%) [26, 30]. Possibly, these people are reluctant to access professional treatment, but it is also possible that they were deprived of help due to unknown barriers [10]. Mild symptoms may progress over time when they remain untreated [10, 17] and in those subjects, the early mild stage of illness may be a window of opportunity to prevent further disease progression.

There are several strengths and limitations of this study that need to be taken into account when interpreting the results. A strength of the study is the longitudinal design. To our knowledge, there is only one international published study that examined determinants of early, delayed and no treatment use in a large representative population study, this study focused on no treatment seeking among people with an alcohol use disorder [34]. This design provided us the opportunity to examine mental health care use in relation to time and course of depression. However, our follow-up period was three years. Thus, there is a possibility that the no treatment users would eventually make contact with professional care in a longer run. Although the sample was nationally representative for the general population, people with insufficient understanding of the Dutch language, who were younger (18–24 years old), had no fixed address and institutionalized people were underrepresented. Furthermore, in the present study we selected only people with a 12-month major depressive disorder. There is a possibility that the onset of the depression might have been before this period. As we do not know the exact service use before the 12 months before baseline, the ‘early treatment users’ may partly exist of actual ‘delayed treatment users’, which may have influenced the results. However, a recent study (2013) showed that the majority of people with depression use mental health care within the first year after the onset of their depression [10]. In addition, it was not possible to examine whether no treatment users were deprived or reluctant to use help. This distinction may be important to examine how we can offer (new) interventions for no treatment users with serious depressive symptoms, who do not receive treatment but may benefit from professional help [26].

Another limitation of this study is the definition of mental health care use. In the present study mental health care use refers to at least one contact made in primary care and or specialized mental health care for emotional or addiction problems. We were not able to examine the nature, intensity or the adequacy and perceived effectiveness of the received treatment. In addition, this study only examined demographic and need factors in relation to mental health care use. Barriers from patients’ perspective were not examined. However, several studies showed that attitudinal beliefs like the desire to handle problems by themselves [15, 48] or the belief that symptoms will abate [14] are frequently mentioned barriers for not using mental health care. Furthermore, supply and organizational aspects of mental health care were not examined. This may have influenced the results since there may be a geographical variation in the pathways to care. However, this is probably more likely for specialized mental health care and not for primary care. Moreover, in the Netherlands, all residents have basic health insurance which covers mental health care costs. Therefore everyone should be able to receive mental heath care, although people might not know that certain therapies are available.

## Conclusion

Most people with a major depressive disorder in the past 12 months accessed mental health care early on (62%). When determining mental health care utilization among people with a major depressive disorder, need factors seem to be important in this process. An important finding is that not everyone from the no treatments users reached remission after three years (11%). In those patients, the mild stage of illness may provide a window of opportunity to prevent further disease progression. Therefore, further research should examine reasons for non-participating in professional care for this specific group.
